# A simple additive staging system for newly diagnosed multiple myeloma

**DOI:** 10.1038/s41408-022-00611-x

**Published:** 2022-01-31

**Authors:** Nadine H. Abdallah, Moritz Binder, S. Vincent Rajkumar, Patricia T. Greipp, Prashant Kapoor, Angela Dispenzieri, Morie A. Gertz, Linda B. Baughn, Martha Q. Lacy, Suzanne R. Hayman, Francis K. Buadi, David Dingli, Ronald S. Go, Yi L. Hwa, Amie L. Fonder, Miriam A. Hobbs, Yi Lin, Nelson Leung, Taxiarchis Kourelis, Rahma Warsame, Mustaqeem A. Siddiqui, Robert A. Kyle, P. Leif Bergsagel, Rafael Fonseca, Rhett P. Ketterling, Shaji K. Kumar

**Affiliations:** 1 Division of Hematology, Mayo Clinic, Rochester, MN USA; 2 Department of Laboratory Medicine and Pathology, Rochester, MN USA; 3 Division of Nephrology and Hypertension, Mayo Clinic, Rochester, MN USA; 4 Division of Hematology, Mayo Clinic, Phoenix, AZ USA

**Keywords:** Myeloma, Risk factors

## Abstract

Risk stratification in multiple myeloma is important for prognostication, patient selection for clinical trials, and comparison of treatment approaches. We developed and validated a staging system that incorporates additional FISH abnormalities not included in the R-ISS and reflects the additive effects of co-occurring high-risk disease features. We first evaluated the prognostic value of predefined cytogenetic and laboratory abnormalities in 2556 Mayo Clinic patients diagnosed between February 2004 and June 2019. We then used data from 1327 patients to develop a risk stratification model and validated this in 502 patients enrolled in the MMRF CoMMpass study. On multivariate analysis, high-risk IgH translocations [risk ratio (RR): 1.7], 1q gain/amplification (RR: 1.4), chromosome17 abnormalities (RR: 1.6), ISS III (RR: 1.7), and elevated LDH (RR: 1.3) were independently associated with decreased overall survival (OS). Among 1327 evaluable patients, OS was 11.0 (95% CI: 9.2–12.6), 7.0 (95% CI: 6.3–9.2), and 4.5 (95% CI: 3.7–5.2) years in patients with 0 (stage I), 1 (stage II), and ≥2 (stage III) high-risk factors, respectively. In the MMRF cohort, median OS was 7.8 (95% CI: NR-NR), 6.0 (95% CI: 5.7-NR), and 4.3 (95% CI: 2.7-NR) years in the 3 groups, respectively (*P* < 0.001). This 5-factor, 3-tier system is easy to implement in practice and improves upon the current R-ISS.

## Introduction

Multiple myeloma (MM) is the second most common hematologic malignancy and is responsible for approximately 2% of all cancer deaths in the United States [1]. With the advent of novel therapeutic agents and drug combinations, survival outcomes have improved considerably [2]. Despite these therapeutic advances, survival outcomes remain highly variable even in uniformly treated clinical trial populations [3, 4]. This disparity highlights the importance of risk stratification at the time of diagnosis for prognostication and patient selection for clinical trials [5, 6]. Several risk stratification systems have been proposed based on clinical characteristics, laboratory studies, bone marrow cytogenetics, and gene expression profiling. Among them, the International Staging System (ISS) and its successor, the revised ISS (R-ISS), have stood the test of time in clinical practice. The ISS was first introduced in 2005 and is based on β2-microglobulin and albumin, which are thought to reflect tumor burden and host status [7]. In clinical practice, the ISS remains a popular choice for risk stratification due to its simplicity, although its ability to discriminate among lower-risk patients is limited in the era of novel therapies [8]. Interphase fluorescence in-situ hybridization (FISH) enabled the identification of high-risk disease independent of ISS stage based on abnormalities such as t(4;14), t(14;16), and del(17p) [9, 10]. The revised ISS (R-ISS) was introduced in 2015 and includes these cytogenetic abnormalities as well as elevated lactate dehydrogenase (LDH) as high-risk markers [11]. The R-ISS has subsequently been validated in several studies [12, 13] and is currently used to risk-stratify patients with newly diagnosed MM [11]. It is thought to perform better than its predecessor in certain patient populations [8, 12, 13]. Recent studies have identified additional cytogenetic abnormalities not included in the R-ISS to be associated with adverse survival outcomes including monosomy 13 [14, 15], gain/amplification of 1q21 [16, 17], and rearrangements involving the *MYC* gene [18–20]. Even though more powerful high-risk classifiers have been developed based on gene expression profiles and cytogenetics, their acceptance in clinical practice remains poor due to the involved resources and need for complex modeling [21–25]. While the presence of high-risk laboratory and cytogenetic features has been incorporated into the R-ISS, the additive effects of multiple co-occurring high-risk disease features have not been accounted for [15, 16]. Importantly, as RISS was developed, assessment of 1q abnormalities was not commonly employed and there was limited data to explore the value of adding this variable. Furthermore, unlike the ISS which had a similar proportion of patients in each of the three stages, R-ISS resulted in majority of patients being classified as intermediate. In light of the identification of additional cytogenetic risk factors and an improved understanding of the prognostic implications of multi-hit disease, we conducted this study to evaluate a new simple additive staging system in patients with newly diagnosed MM [14, 17, 19, 20].

## Patients and methods

### Patients and study design

We included all patients with MM seen in Mayo Clinic in Rochester, Minnesota between 2004 and 2019, who had cytogenetic analysis by FISH performed within 1 year before diagnosis, or within 6 months from the start of first-line treatment. The cohort included 2556 patients 18 years and older, diagnosed with MM between February 2004 and June 2019. Patients were identified from a preexisting database and additional data was obtained by review of electronic medical records. We extracted data on FISH results, standard serum and urine tests, bone marrow biopsy results, first-line treatment, transplant status, and date of death or last follow up. For model development, we included 1327 Mayo Clinic patients who had simultaneous data for all high-risk abnormalities found to be prognostic on multivariate analysis. The validation population included 502 patients enrolled in the MMRF CoMMpass study (MMRF); all data were obtained through the MMRF Researcher Gateway (https://research.themmrf.org). The study was approved by the Mayo Clinic Institutional Review Board. Informed consent was obtained from all patients included in the study.

### Cytogenetics

In the Mayo population, FISH was performed on unsorted bone marrow plasma cells identified by cytoplasmic immunoglobulin staining, as previously described [26]. Only cytoplasmic immunoglobulin positive plasma cells were scored with the goal to reach 50 plasma cells per FISH probe set. The FISH panel included the following probes with their corresponding normal cutoffs (%): 3 centromere (D3Z1, Abbott Molecular) (20%), 7 centromere (D7Z1, Abbott Molecular) (20%), 9 centromere (D9Z1, Abbott Molecular) (20%), 15 centromere (D15Z4, Abbott Molecular) (20%), −13q14 (RB1/LAMP1, Abbott Molecular) (20%), −13q (RB1/LAMP1, Abbott Molecular) (20%), −17p13.1 (TP53/D17Z1, Abbott Molecular) (20%), and −17 (TP53/D17Z1, Abbott Molecular) (20%), enumeration probes.

Dual-color, dual-fusion probes targeting t(11;14) CCND1/IgH (Abbott Molecular) (6%), and break apart probe targeting IgH (in-house developed) (10%) were used. If an IgH rearrangement other than t(11;14) was found by the IgH break apart probe, reflex testing was done using dual-color, dual-fusion probes to identify the translocation partner: t(4;14)(p16.3;q32) FGFR3/IgH (6%), t(14;16)(q32;q23) IgH/MAF (6%), t(14;20)(q32;q12) IgH/MAFB (6%), and t(6;14)(p21;q32) CCND3/IgH (Abbott Molecular) (6%). The t(4;14), t(14;16), and t(14;20) translocations were considered high-risk [5]. Double- and triple hit disease were defined as the presence of a primary high-risk cytogenetic abnormality [t(4;14), t(14;16), t(14;20)] with 1 or 2 additional high-risk cytogenetic abnormalities (chromosome 17 abnormality) and/or 1q gain/amplification), respectively [15]. The *MYC* break apart probe and 1q/1p enumeration probes were introduced for clinical use as part of the Myeloma FISH panel in August 2014. For samples obtained before this date, testing for these cytogenetic abnormalities was performed as an add-on test using samples not subjected to cytoplasmic immunoglobulin counterstain. After this date, testing was performed on plasma cell-enriched samples using the cytoplasmic immunoglobulin stain. The methods used for 1q gain and *MYC* rearrangement testing were previously described [17, 20]. 1q gain was determined using the ratio of 1q22 to 1p (TP73) using an in-house custom developed probe (1q/1p (1q22/TP73) (3.5% for unselected plasma cell samples, 20% for counterstained samples). A break apart probe targeting 8q24.1 (*MYC*, Abbott Molecular) was used to detect a MYC rearrangement (6.5% for unselected plasma cell samples, 10% for counterstained samples). In the validation population, next generation sequencing based FISH (Seq-FISH) was used. This method has been validated and demonstrated comparable specificity and improved sensitivity compared to interphase FISH [27, 28]. Seq-FISH probes were readily available for IGH translocations, MYC rearrangement, 1q gain (20% threshold), and chromosome 17 abnormalities.

### Statistical analysis

Using the entire Mayo cohort (*n* = 2556), we first examined the impact of each of the cytogenetic abnormalities detected by FISH on overall survival (OS) using univariate analysis. These abnormalities included: (1) high-risk translocations involving the immunoglobulin heavy chain (IgH) locus, (2) t(11;14) translocation, (3) trisomies of at least 1 chromosome, (4) rearrangements involving the *MYC* gene locus, (5) gain of 1 or more copies of 1q (1q gain/amplification), (6) monosomy of chromosome 13 (not including patients with 13q deletion alone), and (7) chromosome 17 abnormalities [del(17p) or monosomy of chromosome 17]. Cytogenetic abnormalities significantly associated with survival in the univariate model were included in a multivariate model; those significantly associated with survival in the multivariate model were then included in a final multivariate model with ISS stage III (vs I/II) and elevated LDH. Values above the upper limit of normal of the reporting laboratory were considered elevated for lactate dehydrogenase (above 222 U/l for the Mayo Clinic Laboratories). ISS stages were defined as: β2-microglobulin <3.5 mg/dL and albumin ≥3.5 g/dL (stage I); β2-microglobulin ≥5.5 regardless of albumin (stage III); all other cases were considered stage II [7]. For R-ISS, stage I included patients with ISS stage I, normal LDH, and absence of high-risk cytogenetics; R-ISS stage III included patients with ISS stage III, elevated LDH and/or high-risk cytogenetics; all other cases were considered stage II [11]. Overall survival (OS) was defined as the time from diagnosis of multiple myeloma to death from any cause. Progression-free survival (PFS) was defined as the time from the start of first-line treatment to first disease progression or death from any cause (whichever occurred first). Patients without an event at the end of follow-up were censored. Risk ratios (RR) were calculated based on the univariate and multivariate cox proportional hazards models.

### Model development and validation—The Mayo Additive Staging System (MASS)

First, we updated the R-ISS such that the definition of HR cytogenetic abnormalities included all the FISH abnormalities found to be significantly associated with survival in our final multivariate model. We then compared the PFS and OS of patients based on the updated R-ISS. Next, given that the R-ISS does not give similar weight to all the prognostic factors, we explored a simpler approach that incorporates all the independent prognostic factors, and grouped patients by virtue of the number of risk factors. For this analysis, we included 1327 Mayo Clinic patients who had simultaneous data available for all the variables significantly associated with survival in the final multivariate model. We examined PFS and OS of patients based on the number of high-risk abnormalities; patients with 0, 1, or ≥2 HR abnormalities were considered as stage I, II, or III, respectively. We then performed a subgroup analysis for OS based on age group (<65 years vs. ≥65 years), transplant status, and across 2 time periods (before and after 2012). Next, we evaluated the performance of this model in an independent population of 502 patients enrolled in the MMRF CoMMpass study (MMRF). The performance of the model was measured by Harrell’s Concordance Index (C) [29, 30]. Overall and progression-free survival estimates were calculated using the Kaplan-Meier method, and survival was compared between groups using the Log-rank test [31]. For all tests, 2-sided P values <0.05 were considered statistically significant. Statistical analyses were performed using the JMP (SAS, Cary, NC) and Stata statistical softwares.

## Results

### Baseline patient characteristics

The Mayo clinic cohort included 2556 patients diagnosed with MM between February 2004 and June 2019. The median age was 64 years and 62% were males. The clinical characteristics of patients and the first-line treatments are included in Table 1. Among all patients, 143 (6%) had no abnormality detected by FISH using the probes tested. A trisomy of at least one chromosome was found in 55% of patients tested. t(11;14) was the most common IgH translocation, found in 21%; t(4;14) and t(14;16) were found in 10% and 4%, respectively. A gain of 1 or more copies of chromosome 1q was detected in 31%, and a rearrangement involving the *MYC* gene locus was found in 9%. A monosomy of chromosome 13 was seen in 37%, and an abnormality in chromosome 17 [del(17p)/monosomy 17] was seen in 13%. These results are presented in Table 2.

**Table 1:** Baseline characteristics.

Baseline characteristics	Median (interquartile range)	*N* (%)
**Age (years)**	64 (57–71)	
**Male**		1582 (62)
**ECOG PS** ≥ **2 (vs 0**–**1)**		141 (20)
**Hemoglobin (g/dL)**	11.0 (9.5–12.5)	
**Hemoglobin** ≤ **10** **g/dL**		720 (33)
**Platelets** (×**10**^**9**^**/L)**	210 (162–262)	
**Creatinine (mg/dL)**	1.1 (0.9–1.5)	
**Creatinine** ≥ **2** **mg/dL**		319 (16)
**LDH** > **ULN (units/L)**		298 (17)
**B2M (µg/ml)**	4.0 (2.7–6.6)	
**B2M** > **5.5 vs. (≤5.5)**		701 (32)
**Albumin (g/dL)**	3.6 (3.2–3.9)	
**Albumin** ≤ **3.5 (vs. >3.5)**		928 (48)
**Calcium (mg/dL)**	9.5 (9.0–10.1)	
**Calcium** ≥ **11** **mg/dL**		215 (11)
**Serum M spike (g/dL)**	2.5 (0.7–3.9)	
**Urine M spike (g/24** **h)**	0.05 (0–0.50)	
**Urine albumin (g/24** **h)**	0.05 (0.02–0.14)	
**IgA MM**		464 (25)
**IgG MM**		1177 (62)
**LC MM**		214 (11)
**Involved LC**		
Kappa		1252 (65)
Lambda		674 (35)
**ISS Stage III (vs. I&II)**		710 (33)
**BMPCs (%)**	50 (30–70)	
**PCLI (%)**	0.8 (0.3–1.5)	
**First-line treatment**		
PI		727 (31)
IMiD		720 (31)
PI + IMiD		804 (34)
Other		107 (5)
**Transplant**		1399 (55)
Early (≤1 year from diagnosis)		1184 (85)
Late (>1 year from diagnosis)		215 (15)

Clinical and laboratory characteristics at diagnosis of patients diagnosed with multiple myeloma included in the study. The median (range) are presented for continuous variables and number (percentage) for categorical variables.

*B2M* beta2microglobulin, *BMPCs* bone marrow plasma cells, *IMiD* immunomodulatory drug, *ISS* international staging system, *LC* light chain, *LDH* lactate dehydrogenase, *MM* multiple myeloma, *PCLI* plasma cell labeling index, *PI* proteasome inhibitor, *PS* performance status, *ULN* upper limit of normal.

**Table 2:** Cytogenetic abnormalities in multiple myeloma patients.

Primary abnormalities	Tested *N*	Abnormality *N* (%)
**IgH translocations**		
t(4;14)	2519	248 (10)
t(14;16)	2517	99 (4)
t(11;14)	2522	519 (21)
**Trisomies**	2491	1374 (55)
**Secondary abnormalities**		
**1q gain/amplification**	1896	585 (31)
**Chromosome 17 abnormality (17pdel/monosomy 17)**	2499	337 (13)
**Monosomy 13**	2513	926 (37)
***MYC*** **rearrangement**	1856	160 (9)

Prevalence of recurrent primary and secondary cytogenetic abnormalities in patients tested by FISH at diagnosis.

*IgH* immunoglobulin heavy chain gene locus, *del* deletion.

### Univariate and multivariate survival analysis

The median follow-up in the entire cohort was 6.2 (95% CI: 5.9–6.5) years. At the time of analysis, 58% of patients were alive; the median OS was 7.5 (95% CI: 7.0–8.1) years. On univariate analysis, high-risk IgH translocations (RR: 2.0), *MYC* rearrangements (RR: 1.5), 1q gain/amplification (RR: 1.8), monosomy of chromosome 13 (RR: 1.4), and chromosome 17 abnormalities (RR: 2.0), were all associated with increased risk of death, while the presence of trisomies was associated with decreased risk of death (RR: 0.8); t(11;14) was not prognostic for OS (RR: 1.0). On multivariate analysis including all FISH abnormalities significantly associated with OS on univariate analysis, monosomy 13 (RR: 1.1, *P* = 0.20) and trisomies (RR: 0.9, *P* = 0.08) were no longer prognostic for OS. ISS III and elevated LDH were both associated with increased risk of death on univariate analysis, so these were included in a final multivariate model with high-risk IgH translocations, *MYC* rearrangements, 1q gain/amplification, and chromosome 17 abnormalities. HR IgH translocations (RR: 1.7, *P* < 0.001), 1q gain/amplification (RR: 1.4, *P* < 0.001), chromosome 17 abnormalities (RR: 1.6, *P* < 0.001), ISS III (RR: 1.7, *P* < 0.001), and elevated LDH (RR: 1.3, *P* = 0.01) were all independently associated with decreased OS in the final multivariate model. *MYC* rearrangements were associated with decreased survival, but this was not statistically significant (RR: 1.3, *P* = 0.06). These results are presented in Table 3.

**Table 3:** Univariate and multivariate survival models.

Variable	Univariate		Multivariate (FISH abnormalities only)		Multivariate (all)	
	OS RR (95% CI)	*P* value	OS RR (95% CI)	*P* value	OS RR (95% CI)	*P* value
**HR IgH translocations**	2.0 (1.7–2.3)	**<0.001**	1.6 (1.3–1.9)	**<0.001**	1.7 (1.3–2.1)	**<0.001**
**t(11;14)**	1.0 (0.8–1.2)	0.88	—	—	—	—
**Trisomies**	0.8 (0.7–0.9)	**0.003**	0.9 (0.7–1.0)	0.08	—	—
***MYC*** **rearrangement**	1.5 (1.2–1.9)	**<0.001**	1.5 (1.2–1.9)	**0.002**	1.3 (1.0–1.8)	0.06
**1q gain/amplification**	1.8 (1.6–2.2)	**<0.001**	1.6 (1.4–2.0)	**<0.001**	1.4 (1.2–1.8)	**<0.001**
**Monosomy 13**	1.4 (1.2–1.6)	**<0.001**	1.1 (0.9–1.3)	0.20	—	—
**Del(17p)/monosomy 17**	2.0 (1.7–2.3)	**<0.001**	1.9 (1.6–2.3)	**<0.001**	1.6 (1.3–2.0)	**<0.001**
**ISS III (vs. ISS I/II)**	1.9 (1.7–2.2)	**<0.001**	—	—	1.7 (1.4–2.0)	**<0.001**
**Elevated LDH**	1.6 (1.4–1.9)	**0.001**	—	—	1.3 (1.1–1.7)	**0.01**

Univariate and multivariate analysis including cytogenetic abnormalities, ISS stage and LDH.

*Del* deletion, *HR* high-risk, *IgH* immunoglobulin heavy chain gene locus, *ISS* international staging system, *LDH* lactate dehydrogenase, *OS* overall survival, *RR* risk ratio. Bolded numbers represent *P* values < 0.05.

### PFS and OS based on the updated R-ISS

**Fig. 1:**
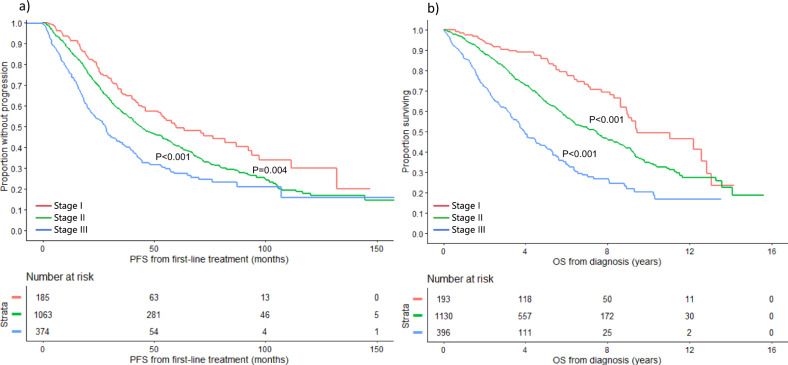
PFS and OS based on the updated R-ISS. **a** PFS (months) and **b** OS (years) in MM patients with stage I (red curve), II (green), and III (blue curve) based on the updated R-ISS. *MM* multiple myeloma, *OS* overall survival, *PFS* progression-free survival, *R-ISS* revised international staging system. The *P* values for each pair of groups are presented between the corresponding curves.

The R-ISS was updated to include 1q gain/amplification in the definition of high-risk cytogenetic abnormalities, in addition to high-risk IgH translocations and chromosome 17 abnormalities. Based on this definition, 193 (11%), 1130 (66%), and 396 (23%) patients had stage I, II, and III disease, respectively. The median PFS was 60.0 (95% CI: 46.1–87.1), 44.0 (95% CI: 40.5–48.8), and 28.1 (95% CI: 23.0–31.4) months in the 3 groups, respectively (*P* < 0.001) (Fig. 1a). Median OS was 9.4 (95% CI: 8.9–12.8), 7.5 (95% CI: 6.4–8.0), and 3.9 (95% CI: 3.6–4.6) years in patients with stage I, II, and III disease, respectively (*P* < 0.001) (Fig. 1b).

### Developing a simpler approach for risk stratification – The MASS

**Fig. 2:**
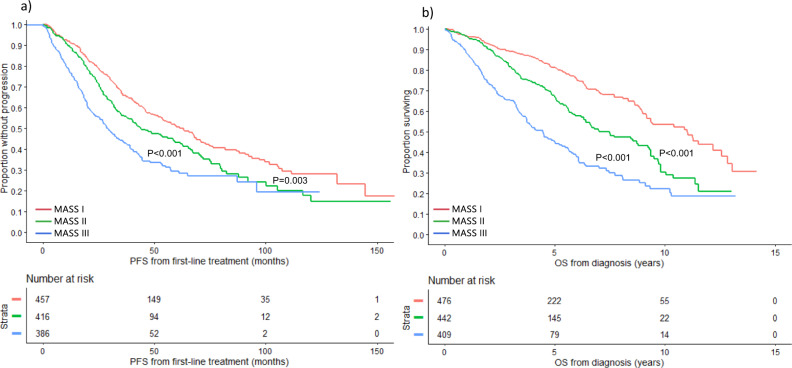
PFS and OS based on the MASS using the Mayo Clinic cohort. **a** PFS (months) and **b** OS (years) in MM patients with no HR factors (stage I) (red curve), 1 HR factor (stage II) (green curve), and ≥2 HR factors (stage III) (blue curve). HR factors are defined as any of: HR IgH translocations, 1q gain/amplification, chromosome 17 abnormality [(del)17p/monosomy 17], ISS stage III, and LDH > ULN. *del*: deletion, *HR*: high-risk, *IgH* immunoglobulin heavy chain gene locus, *ISS* international staging system, *LDH* lactate dehydrogenase, *MASS* Mayo Additive Staging System, *MM* multiple myeloma, *OS* overall survival, *PFS* progression-free survival, *ULN* upper limit of normal. The *P* values for each pair of groups are presented between the corresponding curves.

Among all patients included in the study, 1327 had simultaneous data available for: high-risk IgH translocations, 1q gain/amplification, chromosome 17 abnormalities, ISS stage, and LDH. There were no significant differences in baseline characteristics between evaluable patients and the rest of the cohort (Supplemental Table 1). Among evaluable patients, 476 (36%) had no high-risk factors (stage I), 442 (33%) had 1 high-risk factor (stage II), and 409 (31%) had ≥2 high-risk factors (stage III). Median PFS was 63.1 (95% CI: 53.0–70.8), 44.0 (95% CI: 37.8–58.7), and 28.6 (95% CI: 25.4–34.7) months in the 3 groups, respectively (Fig. 2a). OS was 11.0 (95% CI: 9.2–12.6), 7.0 (95% CI: 6.3–9.2), and 4.5 (95% CI: 3.7–5.2) years in patients with stage I, II, and III disease, respectively (Fig. 2b) (Table 4).

**Table 4:** MASS – Mayo Cohort.

Predictor	Score	Total Score	Stage	PFS (months)	OS (years)
High-risk IGH translocation	+1	0	MASS I	63.1	11.0
1q gain/amplification	+1				
Chromosome 17 abnormality	+1	1	MASS II	44.0	7.0
ISS stage III	+1				
Elevated LDH	+1	2+	MASS III	28.6	4.5

The MASS and associated median progression-free and overall survival estimates for the MAYO population (*n* = 1327).

*IGH* immunoglobulin heavy chain locus, *ISS* International Staging System, *LDH* Lactate Dehydrogenase, *MASS* Mayo Additive Staging System, *OS* overall survival, *PFS* progression-free survival.

### Subgroup analysis by age and transplant status

**Fig. 3:**
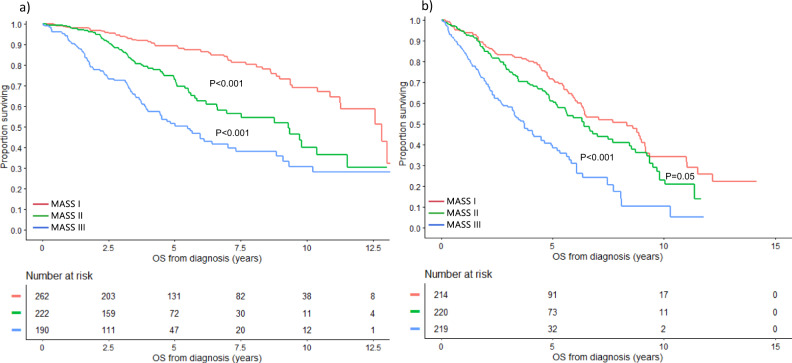
OS based on the MASS by age. OS (years) in MM patients with MASS I (red curve), MASS II (green curve), and MASS III (blue curve) who are **a** <65 and **b** ≥65 years of age. *MASS* Mayo Additive Staging System, *MM* multiple myeloma, *OS* overall survival. The *P* values for each pair of groups are presented between the corresponding curves.

**Fig. 4:**
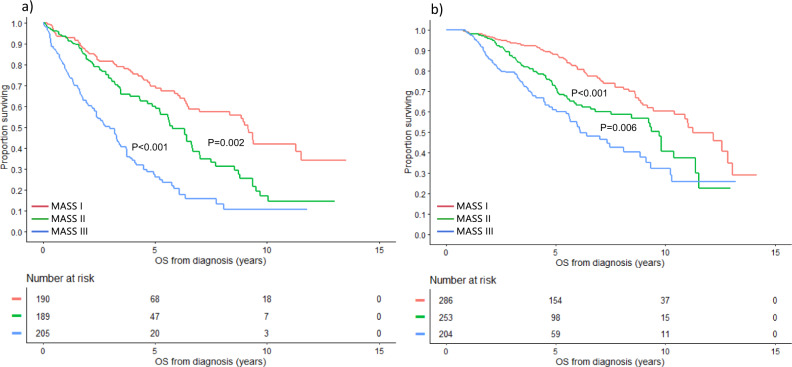
OS based on the MASS by transplant status. OS (years) in MM patients with MASS I (red curve), MASS II (green curve), and MASS III (blue curve) who (**a**) did not undergo transplant and in those who (**b**) underwent transplant. *MASS* Mayo Additive Staging System, *MM* multiple myeloma, *OS* overall survival. The *P* values for each pair of groups are presented between the corresponding curves.

The prognostic ability of this staging system was evaluated based on age group and transplant status. Among patients <65 years, the median OS was 12.8 (95% CI: 11.3–NR), 9.3 (95% CI: 6.6–11.5), and 5.5 (95% CI: 3.9–7.0) years in patients with stage I, II, and III disease, respectively (*P* < 0.001) **(**Fig. 3a). In patients ≥65 years, the median OS was 8.3 (95% CI: 6.3–9.1), 6.4 (95% CI: 5.2–7.7), and 3.7 (95% CI: 3.2–4.5) years in the 3 groups, respectively (*P* < 0.001) (Fig. 3b). Among patients who did not undergo transplant, median OS was 9.1 (95% CI: 6.5–11.5), 5.8 (95% CI: 5.0–6.7), and 3.0 (95% CI: 2.3–3.4) years in patients with stage I, II, and III disease, respectively (*P* < 0.001) (Fig. 4a). In patients who underwent transplant, median OS was 11.3 (95% CI: 10.4–13.0), 9.7 (95% CI: 7.5–10.4), and 6.1 (95% CI: 5.6–8.8) years in the 3 groups, respectively (*P* < 0.001) (Fig. 4b).

### Risk stratification based on the MASS over 2 time periods

Of all the patients included in the study, 1001 were diagnosed with MM prior to the year 2012, including 407 who had available data for staging by the MASS. Among those, 174 (43%), 128 (31%), and 105 (26%) patients had stage I, II, and III disease by MASS, respectively. The median PFS was 36.7 (95% CI: 32.1–46.0), 31.3 (95% CI: 26.6–36.4), 18.8 (95% CI: 13.3–21.8) months in the 3 groups, respectively (*P* < 0.001). The OS in the 3 groups was 10.4 (95% CI: 8.8–11.5), 6.6 (95% CI: 5.5–8.7), and 3.3 (95% CI: 2.3–4.2) years, respectively (*P* < 0.001). Of the 1555 patients diagnosed with MM after year 2012, 920 had available data for MASS staging. Among those, 302 (33%), 314 (34%), and 304 (33%) patients had stage I, II, and III disease by MASS, respectively. The median PFS was NR (95% CI: 74.5-NR), 75.6 (67.4-NR), and 43.4 (95% CI: 37.4–57.5) months in the 3 groups, respectively (*P* < 0.001). The OS was NR (95% CI: NR-7.7-NR), NR (95% CI: 6.3-NR), and 5.7 (95% CI: 4.0–6.1) years, in the 3 groups, respectively, (*P* < 0.001) These results are shown in Supplemental Fig. 1.

On multivariate analysis including the MASS stage, age (≥65 vs. <65), transplant status, and era of diagnosis (prior to vs. after 2012), the MASS stage retained its prognostic ability with statistically different survival differences seen between the stages; the risk ratio (RR) for death was 1.7 for stage II vs. I, 2.0 for stage III vs. II, and 3.3 for stage III vs. I (*P* < 0.001 between all pairs).

### Stage migration using the MASS

**Fig. 5:**
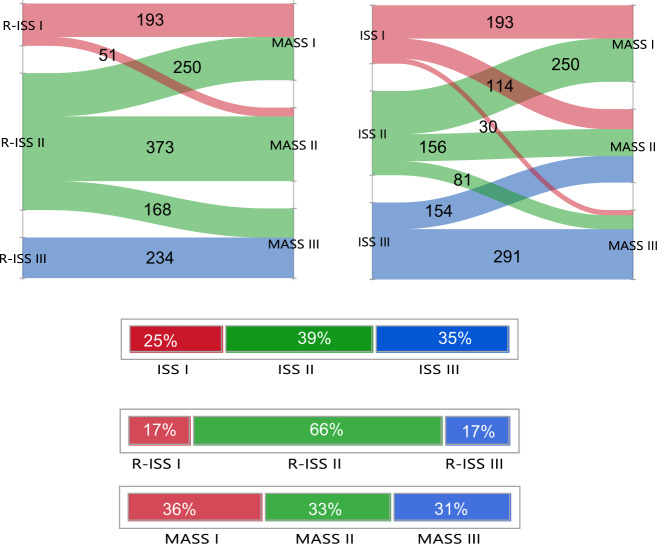
Stage migration between R-ISS and MASS and ISS and MASS. The distribution and migration of patients between disease stages using ISS, R-ISS, and MASS risk stratification systems. *ISS* International Staging System, *R-ISS* Revised International Staging System, *MASS* Mayo Additive Staging System.

Among all patients, 1269 patients had simultaneous data available for R-ISS stage and MASS, including 244 (18%) patients with R-ISS I, 791 (62%) with R-ISS II and 234 (18%) with R-ISS III. Overall, 469 (37%) had stage migration when the MASS was used for risk stratification (Fig. 5). Among R-ISS I patients, 21% were reclassified as stage II using the MASS system; 32% and 21% of patients with R-ISS II were classified as stages I and III, respectively using the MASS system. All patients in R-ISS III were classified as stage III using the MASS system. Among 147 patients with double-hit myeloma, 71 (48%) were classified as stage II using the R-ISS and 76 (52%) were classified as stage III. Among 17 patients with triple hit myeloma, 4 (24%) were classified as R-ISS II and 13 (76%) as R-ISS III. By definition, all patients with double and triple hit MM were classified as stage III using the MASS system. Stage migration from ISS to MASS is also shown in Fig. 5.

Among patients with R-ISS I, patients who were classified as MASS II had inferior OS (median OS: 6.8, 95% CI: 6.3–9.4 years) compared to patients classified as MASS I (median OS: 9.4, 95% CI: 8.9–12.8 years), *P* = 0.03 (supplemental Fig. 2a). Among patients with R-ISS II, median OS was 11.0 (95% CI: 8.9-NR), 7.0 (95% CI: 5.9–9.3), and 4.8 (95% CI: 3.5–5.8) years in patients with MASS I, II, and III, respectively (*P* < 0.001) (supplemental Fig. 2b). Among patients with ISS I, those who were classified as MASS II (median OS: 8.7, 95% CI: 6.3–9.8 years) had decreased OS compared to patients with MASS I (median OS: 9.4, 95% CI: 8.9–12.8 years) (*P* = 0.01), but no significant difference in OS compared to patients with MASS III (median OS: 7.7, 95% CI: 3.3-NR years) (*P* = 0.39) (Supplemental Fig. 2c). Among patients with ISS II, a higher MASS stage was associated with inferior survival; median OS was 11.0 (95% CI: 8.9-NR), 7.5 (95% CI: 5.7–9.5), and 3.9 (95% CI: 3.2–5.7) years in patients with MASS I, II, and III, respectively (*P* < 0.001) (supplemental Fig. 2d).

### Validation using the MMRF cohort

**Fig. 6:**
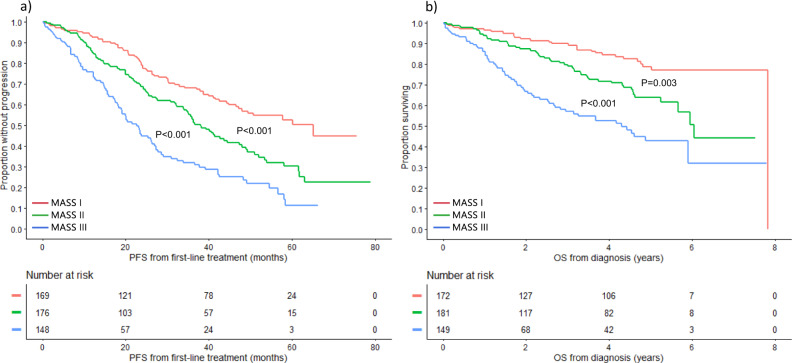
PFS and OS based on the MASS using the MMRF cohort. **a** PFS (months) and **b** OS (years) in MM patients with MASS I (red curve), MASS II (green curve), and MASS III (blue curve) using the MMRF cohort. *MASS* Mayo Additive Staging System, *MM* multiple myeloma, *OS* overall survival, *PFS* progression-free survival. The *P* values for each pair of groups are presented between the corresponding curves.

The MMRF cohort included 502 patients with a median age of 63 (IQR: 56-69) years; 57% were male. Median follow up was 4.4 (95% CI: 4.3-4.6) years. Among all 502 patients, 172 (34%), 181 (36%), and 149 (30%) were classified as stage I, II, and III, respectively. Median PFS was 65.2 (95% CI: 47.2-NR), 38.0 (95% CI: 33.9–44.5), and 22.6 (95% CI: 18.1–26.3) months in patients with stage I, II, and III disease, respectively (*P* < 0.001) (Fig. 6a). Median OS was 7.8 (95% CI: NR-NR), 6.0 (95% CI: 5.7-NR), and 4.3 (95% CI: 2.7-NR) years in the 3 groups, respectively (*P* < 0.001) (Fig. 6b). The Harrell’s C for the MASS was 0.572 (95% CI 0.547–0.598) compared to 0.560 (95% CI: 0.536–0.585) for the R-ISS.

### The MASS as a 4-tier staging system

When the MASS was used as a 4-tier staging system in the Mayo cohort, OS was 11.0 (95% CI: 9.2–12.6), 7.0 (95% CI: 6.3–9.2), 5.0 (95% CI: 4.1–5.8), and 3.4 (95% CI: 2.6–4.1) years in patients with stage I (no risk factors), stage II (1 risk factor), stage III (2 risk factors), and stage IV (≥3 risk factors), respectively (*P* < 0.001). In the MMRF cohort, OS was 7.8 (95% CI: NR-NR), 6.0 (95% CI: 5.7-NR), 4.6 (95% CI: 3.7–5.9), and 1.6 (95% CI: 1.1–3.7) years in patients with stage I, II, III, and IV disease, respectively (*P* < 0.001) (Supplemental Fig. 3). The Harrell’s C for the 4-tier MASS was 0.581 (95% CI 0.554–0.609).

### Impact of the number and type of HR cytogenetic abnormalities

We evaluated the prognosis based on the number of HR cytogenetic abnormalities (HR IgH translocation, 1q gain/amplification, chromosome 17 abnormality (del)17p/monosomy 17, and MYC rearrangement), excluding non-cytogenetic HR parameters of the MASS system (high LDH and ISS III). Median PFS was 57.9 (95% CI: 53.0–63.7), 40.2 (95% CI: 35.2–48.8), 31.1 (95% CI: 26.1–38.1), and 21.9 (95% CI: 15.7–42) months in patients with 0, 1, 2, and ≥3 HR cytogenetic abnormalities (Supplemental Fig. 4a). The corresponding median OS was 10.9 (95% CI: 9.3–11.5), 5.6 (95% CI: 5.2–6.3), 4.8 (95% CI: 3.7–6.1), and 3.1 (95% CI: 2.0–4.0) years, respectively (Supplemental Fig. 4b). Among patients with 1 HR cytogenetic abnormality (610), median OS was 5.6, 5.7, 5.1, and 6.9 in patients with HR IgH translocation, 1q gain/amplification, chromosome 17 abnormality, and *MYC* rearrangement, respectively; having a *MYC* rearrangement alone was associated with better survival compared to having a chromosome 17 abnormality (RR: 0.56, *P* = 0.01) or a HR IgH translocation (RR:0.70, *P* = 0.046), but there was no difference in OS between all other pairs. Among patients with two HR cytogenetic abnormalities (235 patients), there was no difference in OS between patients with one primary and one secondary HR abnormality, (median OS: 4.5, 95% CI: 3.5–6.4 years) and those who had two secondary HR cytogenetic abnormalities (5.7, 95% CI: 2.9–8.1 years) (*P* = 0.77). There was also no difference in OS associated with different combinations of HR cytogenetic abnormalities (global *P* value: 0.66).

## Discussion

Risk stratification in MM remains an important concept both for patient counseling and the development of risk-adapted treatment strategies [5]. While minimal residual disease assessments are emerging as powerful predictors of survival outcomes for patients undergoing treatment, risk stratification in newly diagnosed patients has to rely on pre-treatment patient and disease characteristics [32]. The ISS and its successor, the R-ISS, have prevailed in clinical practice and research alike due to their simplicity and reliance on readily available baseline characteristics [7, 11]. The recognition of additional risk factors motivated the revision of the ISS and resulted in a more powerful predictive model [11]. Since the introduction of the R-ISS, data on additional risk factors including chromosome 13 abnormalities, 1q gains, and *MYC* rearrangements have come to light [15–17, 20]. Furthermore, it has become evident that the co-occurrence of multiple high-risk disease features compounds the risk for adverse outcomes, leading to the concept of double- and triple-hit myeloma [15, 16]. This insight led us to propose a refined additive risk stratification system.

We first evaluated the prognostic impact of known cytogenetic abnormalities in MM. In addition to the high-risk abnormalities included in the R-ISS, 1q gain demonstrated independent prognostic value in our final multivariate model, while the prognostic impact of MYC rearrangements did not reach statistical significance. Trisomies and monosomy 13 were not independently associated with OS when adjusting for other cytogenetic abnormalities, and thus the poor prognostic impact of non-hyperdiploidy [33] and chromosome 13 abnormalities [34–36] may be due to their association with other HR cytogenetic abnormalities. When we used an updated version of the R-ISS, where 1q gain/amplification was included in the definition of high-risk disease, we discriminated 3 groups of patients with significantly different outcomes. However, as with the original R-ISS, most patients had intermediate risk disease (stage II) using this classification (66%). In addition, the R-ISS as originally designed did not consider the compounding effects of co-occurring high-risk disease features. Thus, we proposed a system that stratifies patients based on the number of high-risk factors present at diagnosis: HR IgH translocations, 1q gain/amplification, chromosome 17 abnormalities, ISS III, and/or elevated LDH. Using this system, we discriminated 3 groups of patients with significantly different progression-free and overall survival with nearly a third of the patients distributed between the 3 stages.

The prognostic utility of this system was demonstrated in both age groups: ≥65 years and <65 years, in transplant-eligible and ineligible patients, and over 2 time periods. In addition, its prognostic value was shown in 2 independent populations. Compared to the R-ISS, the 3-tier MASS re-classified approximately one third of patients, underscoring how much our understanding of high-risk disease has been reshaped by the concept of multi-hit disease. Furthermore, the MASS retained its performance and discriminatory ability when used as a 4-tier risk stratification system which can be utilized if additional discrimination among high-risk patients is desired. This application has the potential to serve as an important tool in the design of clinical trials exploring intensification of treatments in high-risk patients. Importantly, this model will lend itself for incorporation of other risk factors as they are identified in the future.

Some limitations of this study include the long period of time over which data were collected during which treatments and transplant-eligibility criteria changed, and the exclusion of non-evaluable patients which may create selection bias. In addition, we did not evaluate some cytogenetic abnormalities for which data were not available like deletion 1p32 which has an estimated prevalence of 7% in newly diagnosed patients and has been associated with worse outcomes [37]. Furthermore, the lack of prognostic value of *MYC* abnormality in this study may be due to the limited number of patients with available cytogenetic data for *MYC*, and thus its added value in risk stratification requires further evaluation in larger studies. The impact of the clonal plasma cell percentage harboring secondary cytogenetic abnormalities and the genetic mutational profile on risk stratification were not assessed in this study and also warrant exploration in future studies.

## Conclusion

In summary, we developed and validated a simple, additive 5-factor 3-tier risk model for newly diagnosed MM in two diverse patient populations that is easy to implement in clinical practice and can play an important role in patient selection for clinical trials.

## Supplementary information

Supplemental Material
