# Supplementary material for: A simple additive staging system for newly diagnosed multiple myeloma
Source: Blood Cancer J. 2022 Jan 31;12(1):21. doi: 10.1038/s41408-022-00611-x (PMC8803917; doi:10.1038/s41408-022-00611-x)
Supplement: Supplementary file 1 — Supplemental Material [file 41408_2022_611_MOESM1_ESM.pdf]

Supplemental Material:

Supplemental table 1: Baseline characteristics of evaluable and non-evaluable patients

Baseline characteristics	Patients included in risk assessment median (IQR) N (%)	Patients not included in risk assessment median (IQR) N (%)	P value
**Age (years)**	64 (57-71)	64 (57-70)	NS
**Age ≥70 (vs. <70)**	403 (30)	343 (28)	NS
**Male**	823 (62)	759 (62)	NS
**ECOG PS ≥2 (vs. 0-1)**	86 (19)	55 (23)	NS
**Hemoglobin (g/dL)**	11.0 (9.4-12.6)	11.0 (9.5-12.5)	NS
**Hemoglobin ≤10 g/dL**	424 (35)	296 (32)	NS
**Creatinine (mg/dL)**	1.0 (0.8-1.4)	1.1 (0.9-1.5)	NS
**Creatinine ≥2 mg/dL**	183 (16)	136 (16)	NS
**LDH (>ULN)**	217 (16)	81 (17)	NS
**B2M (µg/ml)**	4.0 (2.8-6.9)	3.9 (2.7-6.3)	NS
**B2M >5.5 vs. (≤5.5)**	442 (33)	259 (30)	NS
**Albumin (g/dL)**	3.6 (3.3-3.8)	3.6 (3.2-3.9)	NS
**Albumin ≤3.5 (vs. >3.5)**	543 (47)	385 (49)	NS
**Calcium (mg/dL)**	9.6 (9.1-10.1)	9.5 (9.0-10.1)	NS
**Calcium ≥11 mg/dL**	117 (10)	98 (12)	NS
**Serum M spike (g/dL)**	2.6 (0.8-3.9)	2.5 (0.6-3.9)	NS
**Urine M spike (g/24 hrs.)**	0.05 (0-0.53)	0.06 (0-0.5)	NS
**Urine albumin (g/24 hrs.)**	0.06 (0.03-0.15)	0.05 (0.02-0.13)	NS

**ISS Stage III (vs I&II)**	445 (34)	265 (31)	NS
**BMPCs (%)**	50 (30-71)	50 (25-70)	**0.03**
**PCLI (%)**	0.8 (0.3-1.5)	0.8 (0.2-1.6)	NS
**FISH abnormality**			
HR IgH translocation	212 (16)	163 (14)	NS
1q gain/amplification	396 (30)	189 (33)	NS
Ch17 abnormality	185 (14)	152 (13)	NS
MYC abnormality	121 (9)	39 (7)	NS
**First-line treatment**			
PI	386 (31)	341 (31)	-
IMiD	368 (30)	352 (32)	-
PI + IMiD	482 (39)	322 (29)	-
Other	11 (1)	96 (9)	-
**Transplant**	743 (56)	656 (53)	NS

Supplemental table 1: Baseline characteristics. Baseline characteristics at diagnosis of patients included in the risk assessment system compared to the rest of the patients in the cohort. The median (IQR) are presented for continuous variables and number (percentage) for categorical variables. *Abbreviations: B2M: beta2microglobulin, BMPCs: bone marrow plasma cells, Ch17: chromosome 17, HR: high-risk, IgH: immunoglobulin heavy chain gene locus, IMiD: immunomodulatory drug, IQR: interquartile range, ISS: international staging system, LDH: lactate dehydrogenase, NS: not significant, PCLI: plasma cell labeling index, PI: Proteasome inhibitor, PS: performance status. Bolded numbers represent P values <0.05.*

Supplemental figure 1: PFS and OS based on the MASS by time period:

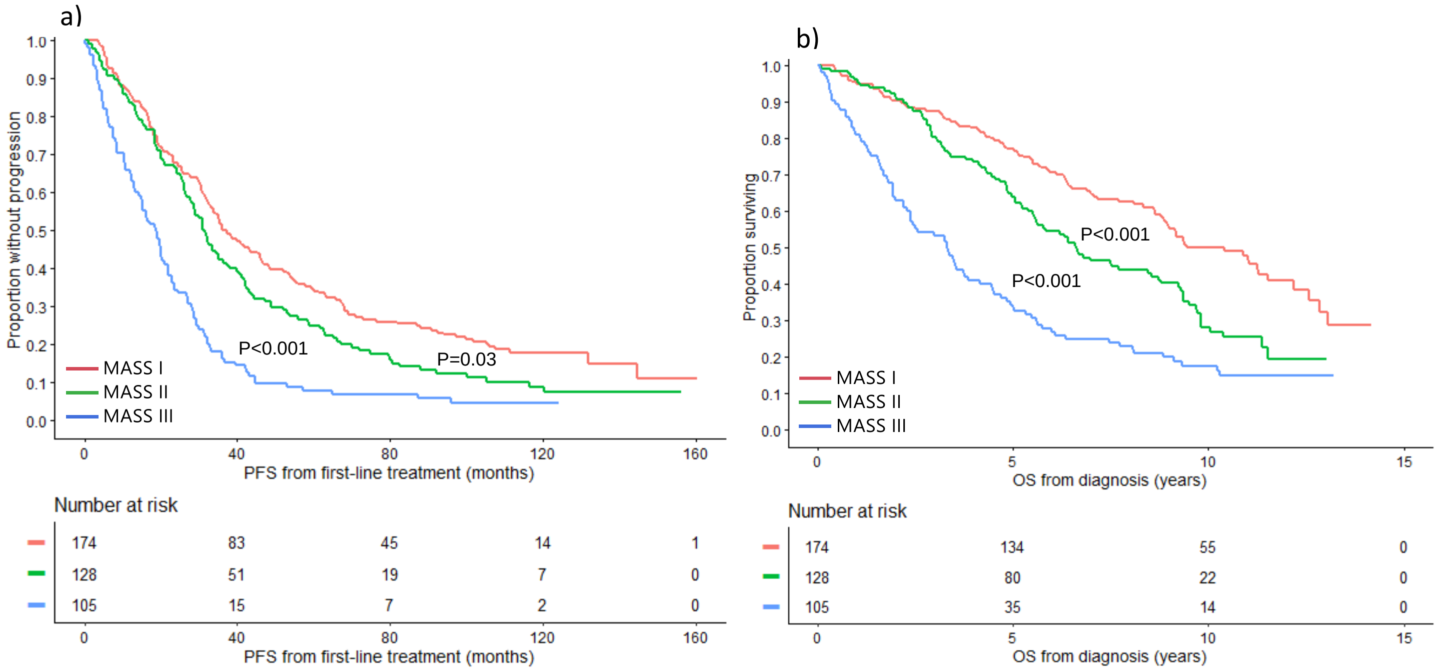

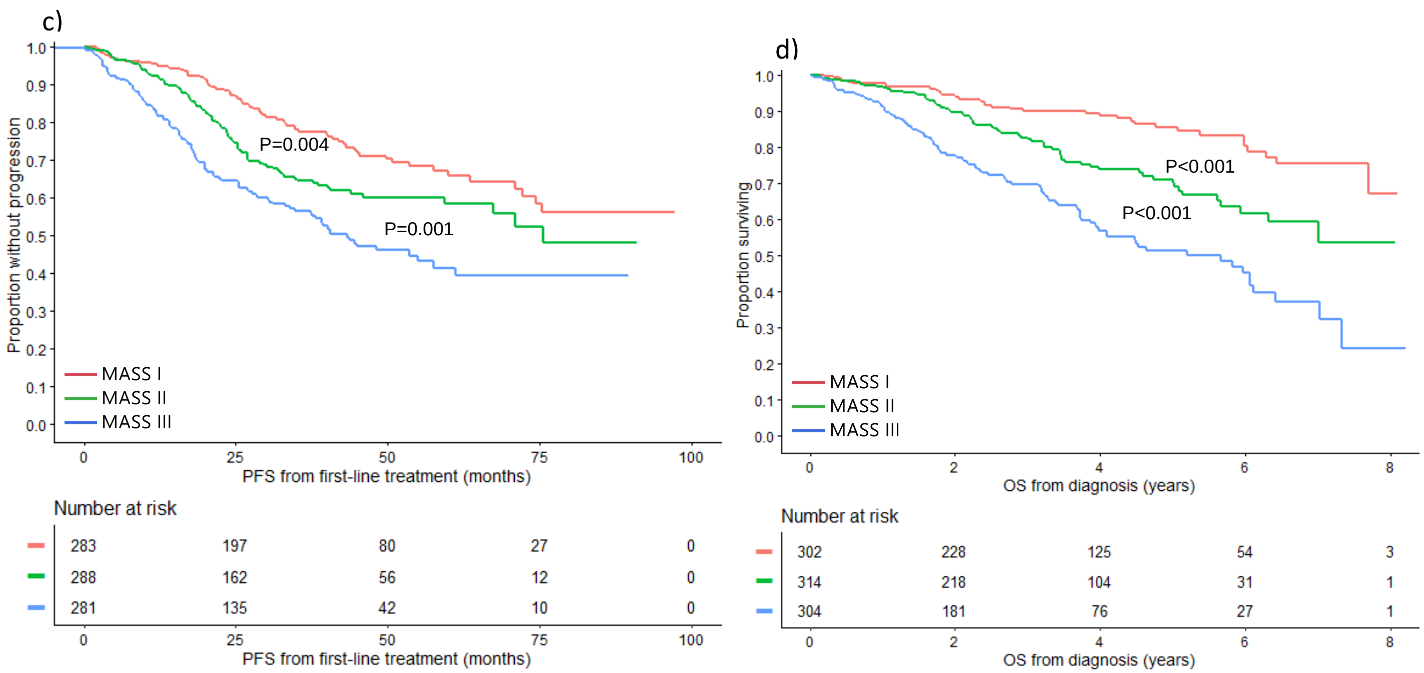

Supplemental figure 1: PFS and OS based on the MASS by time period. PFS (months) in MASS stage I (red curve), stage II (green curve), and stage III (blue curve) among patients with MM diagnosed a) before 2012 and those diagnosed c) after 2012. OS (years) in MASS stage I (red curve), stage II (green curve), and stage III (blue curve) among patients with MM diagnosed b) before 2012 and those diagnosed d) after 2012. *Abbreviations: MASS: Mayo Additive Staging System, MM: multiple myeloma, OS: overall survival, PFS: progression-free survival. The P values for each pair of groups are presented between the corresponding curves.*

Supplemental figure 2:

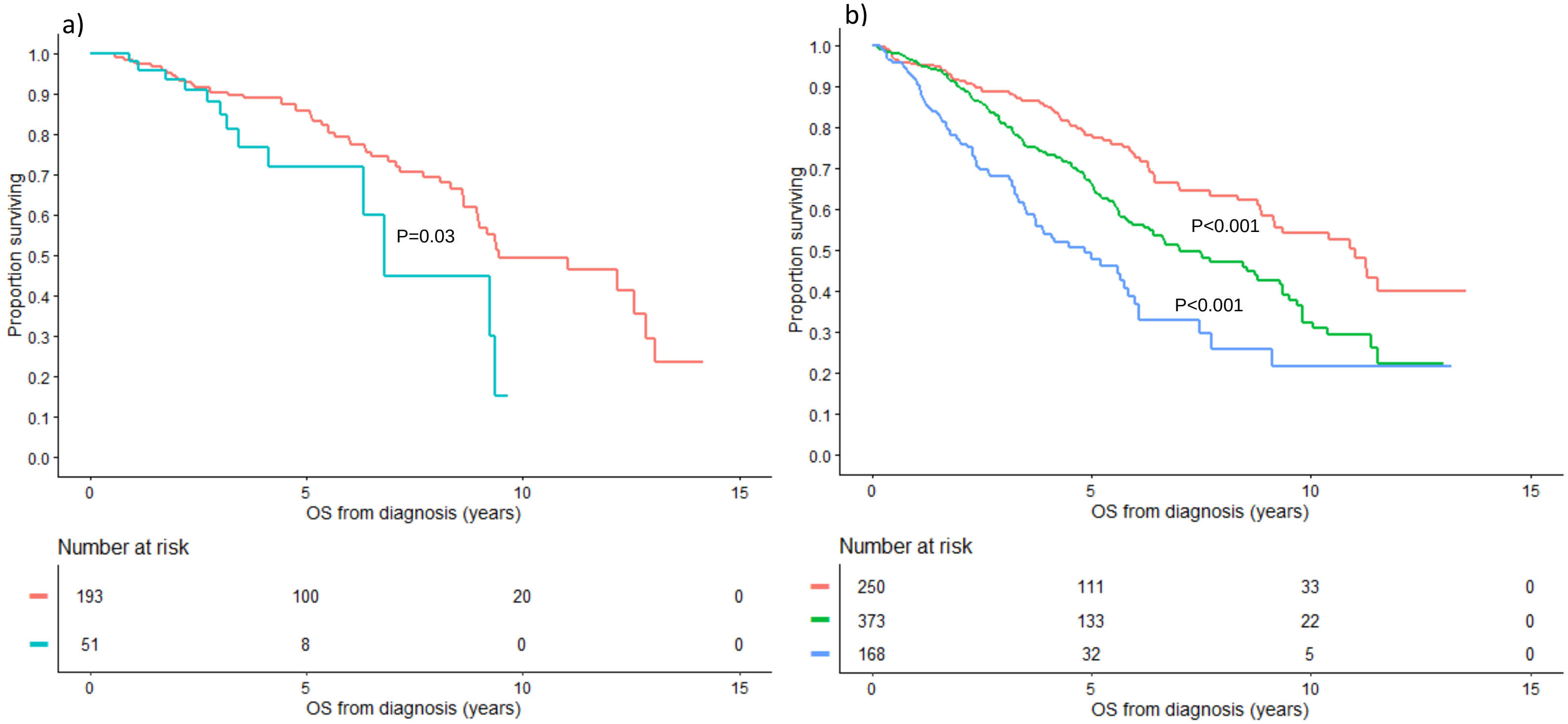

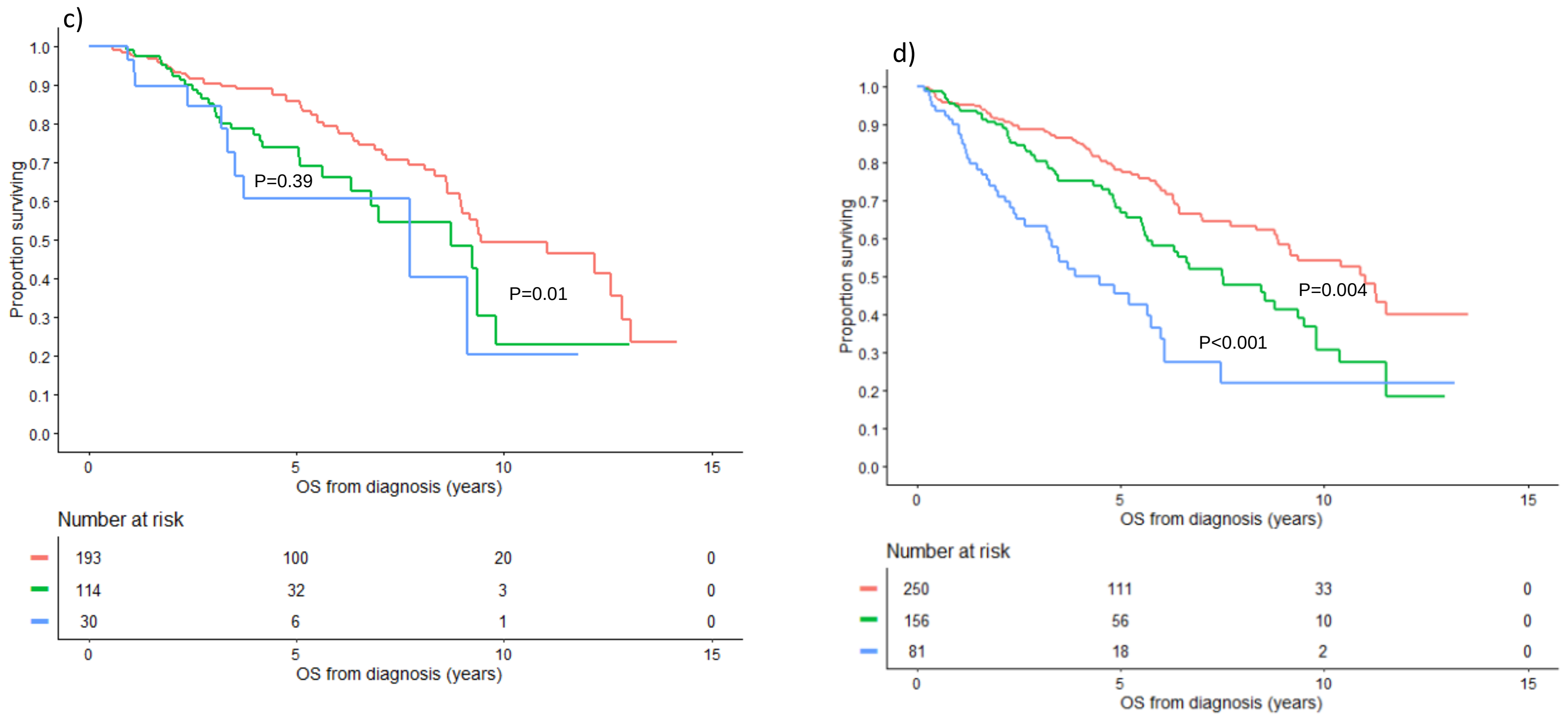

Supplemental figure 2: OS by ISS, R-ISS and MASS. a) OS (years) in MASS stage I (red curve) and stage II (blue curve) among patients with R-ISS I. OS (years) in MASS stage I (red curve), stage II (green curve), and stage III (blue curve) among patients with b) R-ISS II, c) ISS I, and d) ISS II. *Abbreviations: ISS: International Staging System, MASS: Mayo Additive Staging System, MM: multiple myeloma, OS: overall survival, R-ISS: Revised international Staging system. The P values for each pair of groups are presented between the corresponding curves.*

Supplemental figure 3: OS using the MASS as a 4-tier system

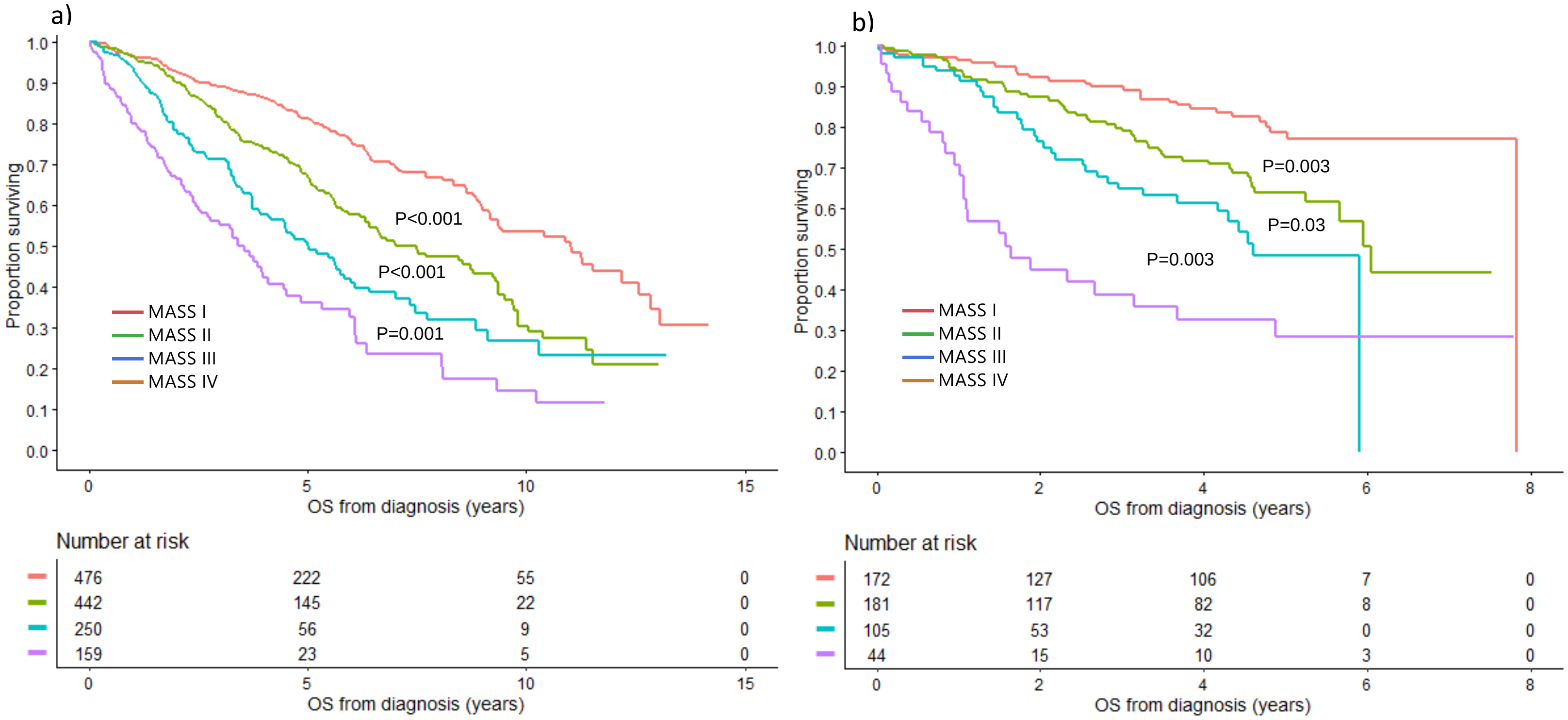

Supplemental figure 3: OS using the MASS as a 4-tier system. OS (years) in MM patients with no HR factors (stage I) (red curve), 1 HR factor (stage II) (green curve), 2 HR factors (stage III) (blue curve), and ≥3 HR factors (stage IV) (yellow curve) in the a) Mayo cohort, and b) MMRF cohort. HR factors are defined as any of: HR IgH translocations, 1q gain/amplification, chromosome 17 abnormality [(del)17p/monosomy 17], ISS stage III, and LDH >ULN. *Abbreviations: del: deletion, HR: high-risk, IgH: immunoglobulin heavy chain gene locus, ISS: international staging system, LDH: lactate dehydrogenase, MM: multiple myeloma, OS: overall survival, ULN: upper limit of normal. The P values for each pair of groups are presented between the corresponding curves.*

Supplemental figure 4:

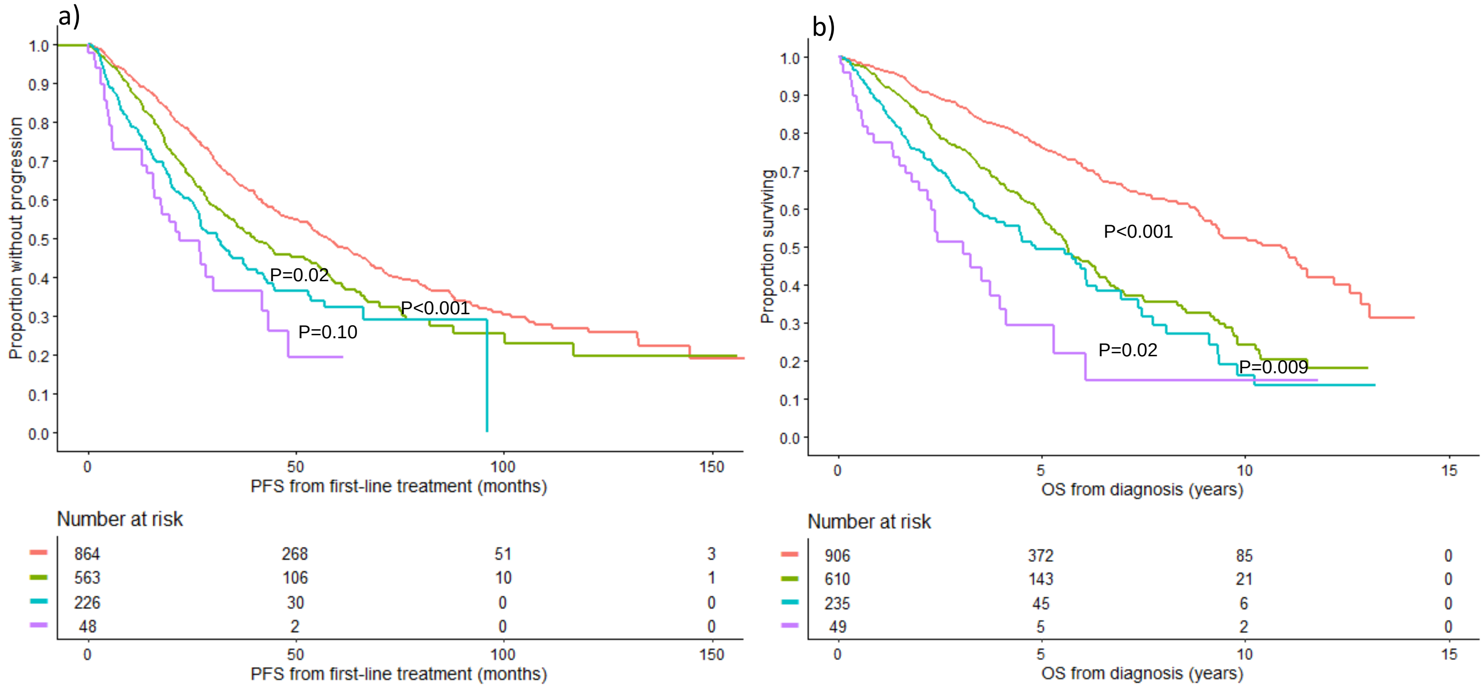

Supplemental figure 4: PFS and OS based on the number of HR FISH abnormalities. a) PFS (months) and b) OS (years) in MM patients with no HR FISH abnormalities (red curve), 1 HR FISH abnormality (green curve), 2 HR FISH abnormalities (blue curve), and ≥3 HR FISH abnormalities (purple curve). HR FISH abnormalities are defined as any of: HR IgH translocation, 1q gain/amplification, chromosome 17 abnormality [(del)17p/monosomy 17], and *MYC* rearrangement. *Abbreviations: del: deletion, HR: high-risk, IgH: immunoglobulin heavy chain gene locus, MM: multiple myeloma, OS: overall survival, PFS: progression-free survival. The P values for each pair of groups are presented between the corresponding curves.*
